# Global patterns in anaphylaxis due to specific foods: A systematic review

**DOI:** 10.1016/j.jaci.2021.03.048

**Published:** 2021-12

**Authors:** Alessia Baseggio Conrado, Nandinee Patel, Paul J. Turner

**Affiliations:** a National Heart & Lung Institute, Imperial College London, London, United Kingdom; b Department of Paediatrics and Child Health, University of Sydney, Sydney, Australia

**Keywords:** Allergen labeling, anaphylaxis, Codex, epidemiology, food allergy, prevalence, LTP, Lipid transfer protein, NASWP, North America/Southwest Pacific

## Abstract

**Background:**

There are increasing global data relating to prevalence of food allergy and food-induced anaphylaxis; however, this is often based on surrogate measures of sensitization rather than objective symptoms at food challenge. In terms of protecting food-allergic consumers from reactions, to our knowledge, there has been no global survey assessing geographic differences in the proportion of anaphylaxis triggered by specific foods.

**Objective:**

We sought to identify common triggers for food-induced anaphylaxis and how these vary from country to country.

**Methods:**

Systematic review of relevant reports published between January 2010 and November 2020. Results were reported following PRISMA guidelines. Publications were screened and data extracted by 2 independent reviewers, and the risk of bias was assessed.

**Results:**

Sixty-five studies (encompassing 41 countries and all 6 regions as defined by the Food and Agriculture Organization of the United Nations) were included. Significant regional variations in the most common triggers of food anaphylaxis were seen; however, in general, there was good agreement between local legislative requirements for allergen disclosure and the most common allergens for each region or nation.

**Conclusions:**

Local legislation for allergen disclosure generally reflects those allergens commonly responsible for food anaphylaxis. Cow’s milk and crustaceans appear to cause a higher proportion of anaphylaxis compared to peanut in some regions.

## Graphical abstract

**Figure:**
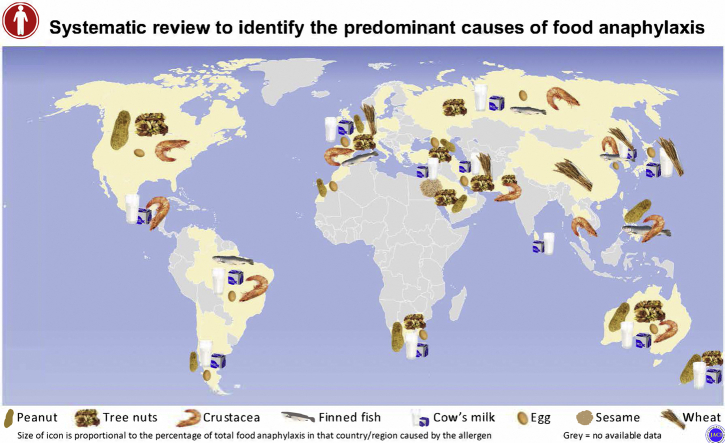

Food supply increasingly involves supply chains across multiple countries. The Codex Alimentarius (often abbreviated to Codex) is a set of international food standards, guidelines, and codes of practice established by the Food and Agricultural Organization of the United Nations and World Health Organization to facilitate the safety of global trade in food supply. Currently, the Codex requires disclosure for ingredients relating to 8 food groups: cereals containing gluten, crustaceans, egg, fish, peanut and soybean, milk, and tree nuts; sulfites (where present at concentrations of ≥10 mg/kg) must also be declared.^1^

The Codex list includes food allergens that are generally considered to cause over 90% of food-induced allergic reactions in most regions. However, anaphylaxis has been reported to almost all foods, and there are significant geographic differences in the prevalence of allergen-specific food allergies worldwide,^2^ presumably as a result of differences in dietary consumption and/or exposure. Some countries/regions therefore include additional allergens that must be declared on food labels.^3^

There are increasing data globally relating to the relative prevalence of food allergy due to specific foods; however, these epidemiologic data may not correspond to the list of foods that commonly cause anaphylaxis.^4^ Prevalence data should ideally be derived from unselected populations, but this often results in very small numbers of individuals allergic to a specific food and thus a high level of uncertainty over the resulting estimated prevalence data generated. More information relating to specific food triggers can be obtained from less rigorous methodologies (for example, diagnosis based on self-report, or the presence of sensitization with or without clinical history). However, this may not correspond to real-world data relating to the occurrence of food-induced allergic reactions due to accidental exposure. This may be because some food allergies resolve over time (for example, the majority of younger children allergic to cow’s milk and hen’s egg), or because some allergens (such as those implicated in pollen–food allergy syndrome) are not generally considered to cause systemic reactions in most affected individuals.^5^ In terms of assessing the risk posed to food-allergic consumers, to our knowledge, there has been no global survey assessing geographic differences in the relative proportions of anaphylaxis due to specific foods. We therefore undertook a systematic review to address this evidence gap.

## Methods

We undertook a systematic review of the literature to identify studies reporting proportions of anaphylaxis in different countries/regions due to specific food triggers. This was undertaken and reported in accordance with the PRISMA statement.^6^

### Search strategy

We used the search strategy from a systematic review of global anaphylaxis epidemiology^4^ (but limited to food allergens as the trigger for anaphylaxis) to perform a systematic search on the following electronic databases: Medline (Ovid), PubMed, and Embase (Ovid). There was no registered protocol for this review, but the methods and analyses were planned a priori*.* No language restrictions were made, and we planned to include non-English papers if they met our inclusion criteria. Abstracts were independently screened by 2 authors, and disagreements were resolved by discussion. We also reviewed reference lists of included studies and review articles to identify other relevant studies.

### Study selection

We included all studies that provided details as to specific triggers for food anaphylaxis, either cases in patients presenting to a medical facility or reported to a central registry. We also included case series recording more than 10 fatalities due to food anaphylaxis. Risk of bias was assessed according to Hoy et al.^7^ Studies at high risk of bias were excluded unless there were no other data sets to provide information for that specific country. Where multiple publications were identified for the same data set with overlapping time periods, we included the report with the largest number of individuals where we could be certain that no duplication was present.

### Data extraction and analyses

Data were extracted in duplicate, and any discrepancies identified were resolved by discussion and/or by contacting authors for clarifications. The different definitions used for anaphylaxis in individual studies were noted accordingly, along with an indication of the completeness of the data (proportion of cases where a specific food trigger was identified). Data were expressed as the proportion of anaphylaxis cases due to a specified food trigger compared to all cases of food anaphylaxis reported in that case series. Heat maps were used to identify the most common food allergens in each data series and to facilitate between-country comparisons.

In order to compare the proportion of anaphylaxis to reported prevalence for that allergen by region, both prevalence rates and anaphylaxis frequencies for individual allergens were pooled across studies using a generalized linear mixed model in R 4.0.3 software (R Foundation for Statistical Computing, Vienna, Austria; https://www.r-project.org/) (metaprop function, metafor package, logit transformation with a random intercept logistic regression model for the summary estimate). This approach avoids many of the issues surrounding the use of transformations when undertaking meta-analyses of proportions.^8^^,^^9^ We conducted meta-analyses even if significant heterogeneity was seen between study estimates, as is the norm when conducting meta-analysis of proportions. Additional information regarding the data sets used to determine prevalence is available in the Online Repository at www.jacionline.org and Table E1.

## Results

**Fig 1:**
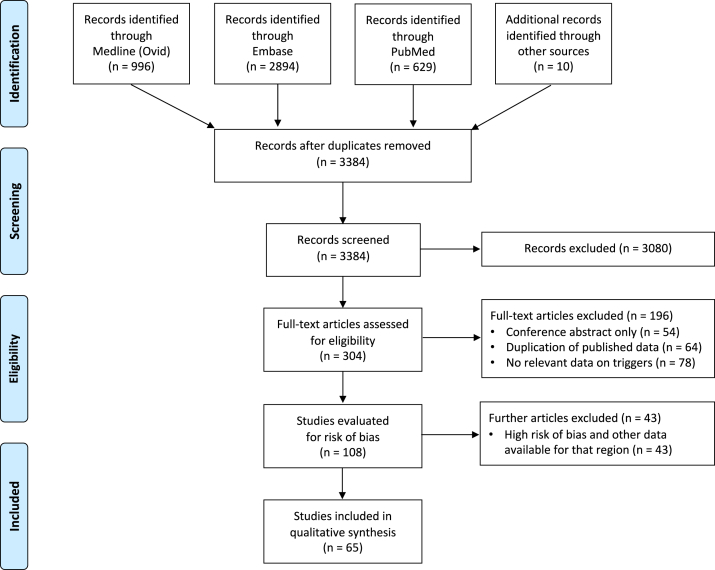
PRISMA flow diagram.

**Fig 2:**
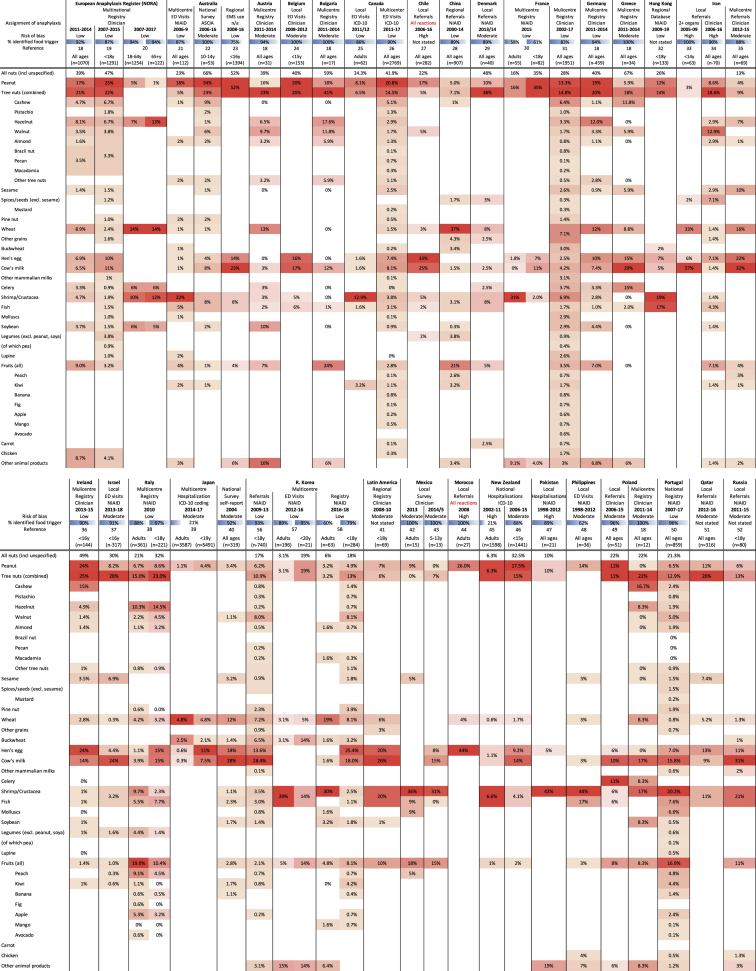

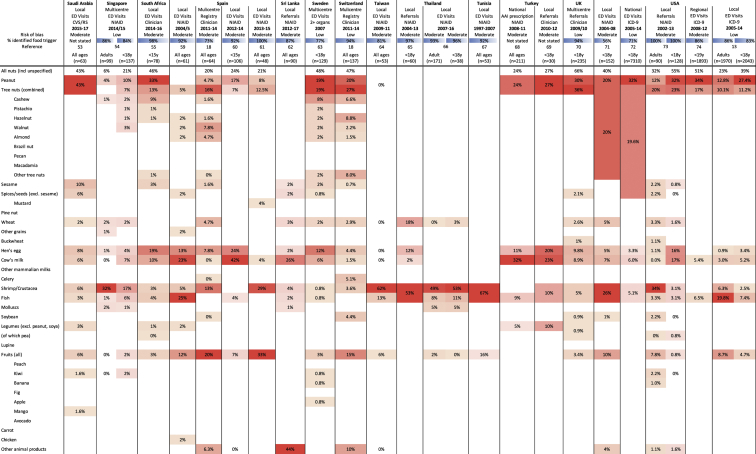
Studies reporting food anaphylaxis events presenting to medical facilities (ie, emergency department [ED] visits, hospitalizations, clinics). Data are presented as the proportion of all reported cases of food anaphylaxis due to the specified food trigger. Heat map colors indicate relative (rather than absolute) prevalence of specific foods within each case series.

**Fig E1:**
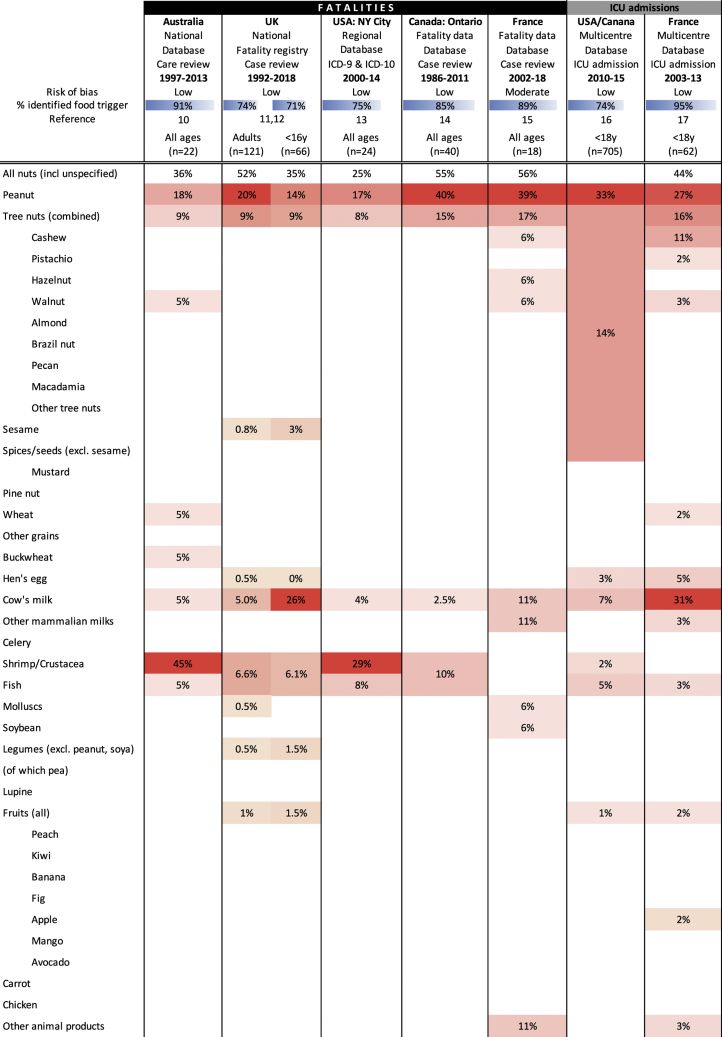
Studies reporting fatalities and intensive care unit (ICU) admissions for food anaphylaxis. Shown is the percentage of all cases attributed to a food allergen caused by a specified food trigger. Heat map colors indicate relative (rather than absolute) prevalence of specific foods within each case series.

Sixty-five studies (encompassing 41 countries and all 6 regions as defined by the Food and Agriculture Organization of the United Nations) were identified for inclusion (Fig 1). Details of the individual studies appear in Fig E1 in the Online Repository at www.jacionline.org and Fig 2, along with the definition of anaphylaxis used and an indication of data completeness and risk of bias assessment.

In total, 6 studies reported food anaphylaxis fatalities (covering Australia,^10^ the United Kingdom,^11^^,^^12^ the United States [New York City],^13^ Canada [Ontario],^14^ and France^15^), while an additional 2 studies reported intensive care admissions due to food-induced anaphylaxis.^16^^,^^17^ These studies are reported in Fig E1. The most common triggers reported for severe reactions were peanut, tree nuts, cow's milk and crustaceans. Fifty-seven other studies were included: 10 reports from anaphylaxis registries, 21 reporting visits to emergency departments, and 4 reporting hospitalizations due to food anaphylaxis, 4 surveys, 1 report of emergency medical services usage, and 17 describing clinic referrals for food anaphylaxis. All but 2 studies provided details regarding the specific triggers for food anaphylaxis; 2 (one from Chile, another from Morocco) included non-anaphylaxis reactions, but they were included in this analysis as a result of an absence of alternative data for these countries. These studies are reported in Fig 2.^18^, ^19^, ^20^, ^21^, ^22^, ^23^, ^24^, ^25^, ^26^, ^27^, ^28^, ^29^, ^30^, ^31^, ^32^, ^33^, ^34^, ^35^, ^36^, ^37^, ^38^, ^39^, ^40^, ^41^, ^42^, ^43^, ^44^, ^45^, ^46^, ^47^, ^48^, ^49^, ^50^, ^51^, ^52^, ^53^, ^54^, ^55^, ^56^, ^57^, ^58^, ^59^, ^60^, ^61^, ^62^, ^63^, ^64^, ^65^, ^66^, ^67^, ^68^, ^69^, ^70^, ^71^, ^72^, ^73^, ^74^

### Major causes of food-induced anaphylaxis by Codex region

**Fig 3:**
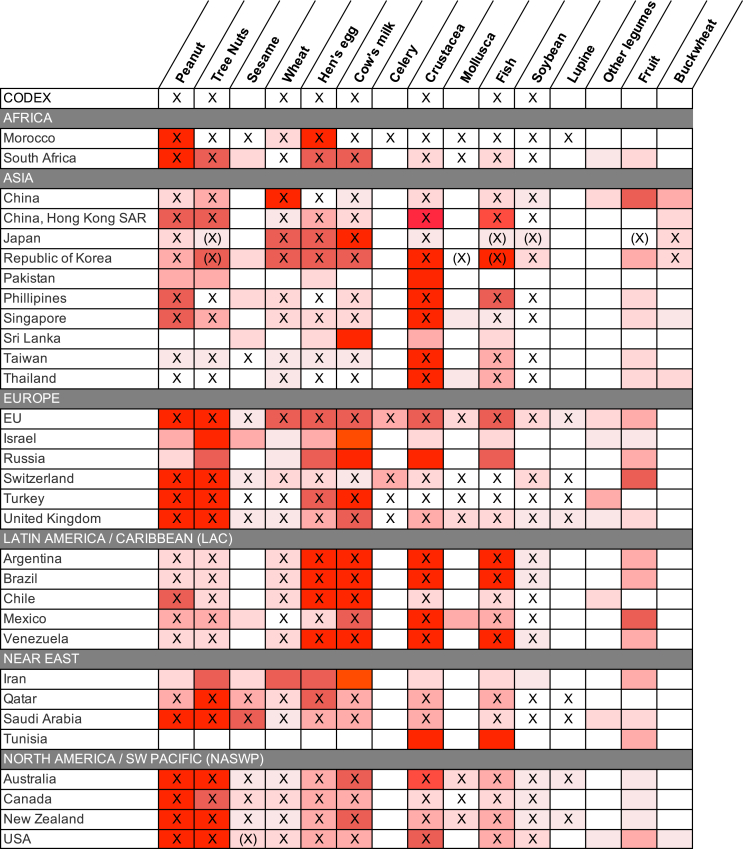
Common food allergens reported to cause anaphylaxis by Codex Alimentarius (Codex) region and country. X indicates local legislation requiring disclosure for that allergen; (X), more limited or voluntary disclosure recommended.^3^ Heat map colors indicate relative (rather than absolute) prevalence of that allergen (group) as a common cause of food anaphylaxis in that region.

**Fig 4:**
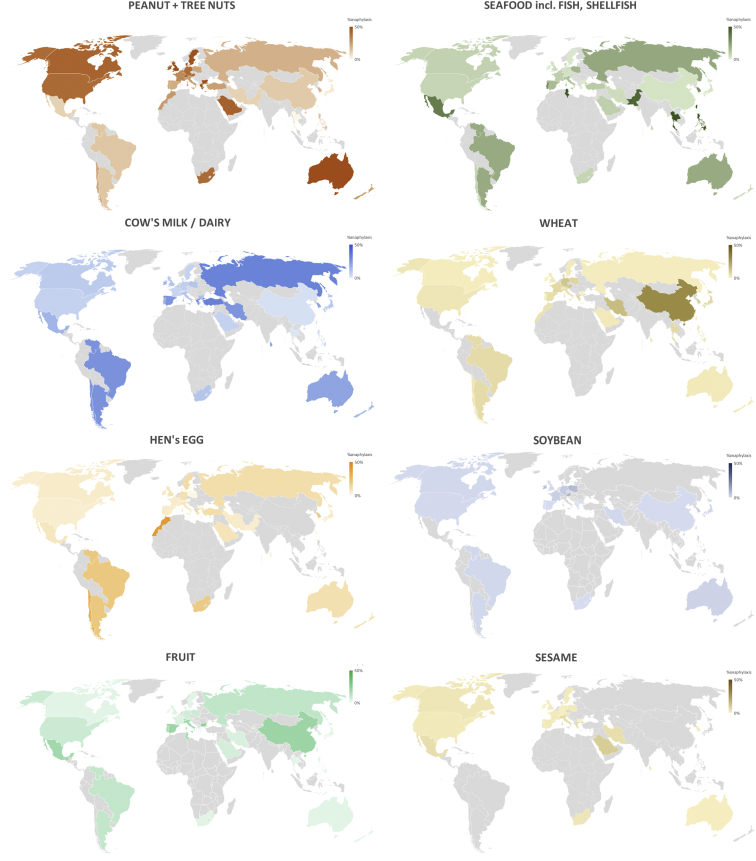
Global maps showing variations in the relative proportion of reported food anaphylaxis cases due to a specific food trigger (peanut and tree nuts [combined], seafood, cow’s milk, wheat, egg, soybean, fruit [combined] and sesame), by country.

To further assess geographic variations in the most common food allergens reported to cause anaphylaxis, the data from Fig 2 were tabulated by Codex region (Fig 3) and plotted on a global map (Fig 4). These data demonstrated that while there are some allergens that are a common cause of anaphylaxis in multiple regions, there are also some foods that seem to be limited as a common trigger to just 1 or 2 regions. Of note, soya was not a major cause of food anaphylaxis in any region.

### Common food triggers for anaphylaxis compared to prevalence

**Fig 5:**
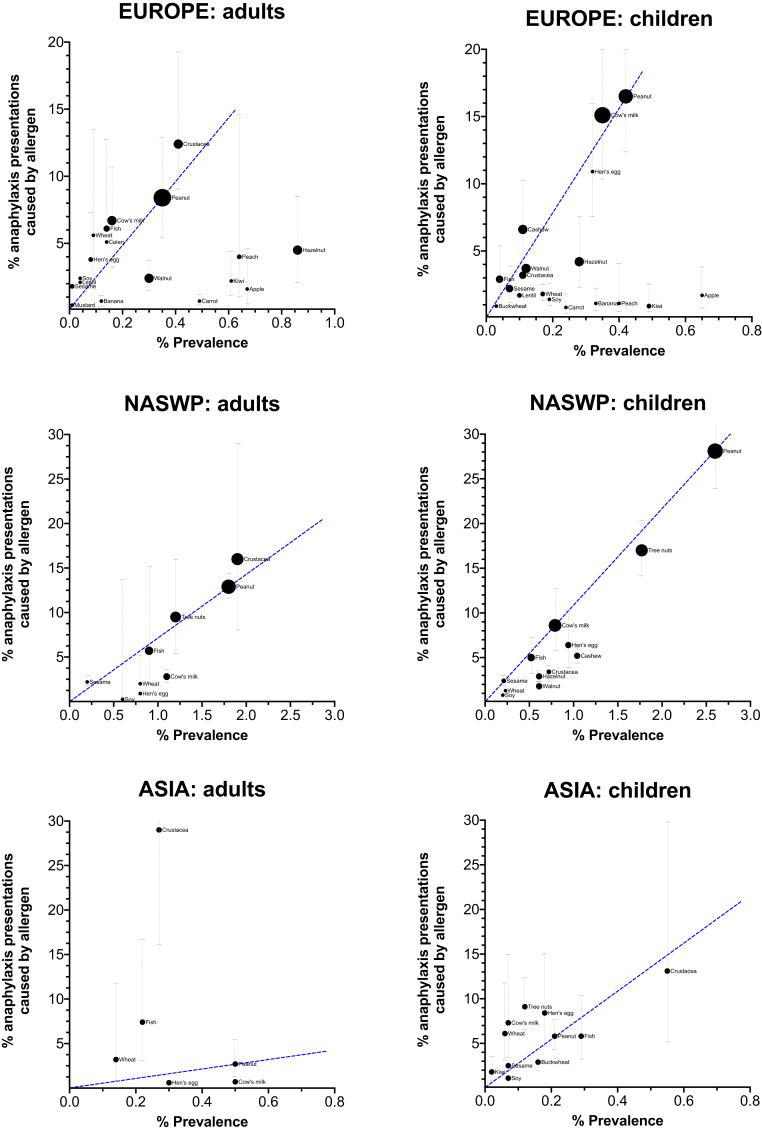
Comparison of the proportion of total food anaphylaxis caused by a specific food trigger in any given region compared to its prevalence as a cause of food allergy. *Dotted lines* represent 95% confidence interval (CI). The 95% CIs for prevalence estimates are reported in Table E1 in the Online Repository at www.jacionline.org. For NASWP, the *bubble size* represents the relative number of fatalities reported due to food anaphylaxis for the specific food trigger. (These data were not available for the Asia region.) The *blue dashed line* is included to facilitate comparisons of these data to peanut.

Finally, prevalence data were obtained for Europe, North America/Southwest Pacific (NASWP), and Asia from the literature, and the estimated pooled prevalence (derived from meta-analysis, and reported in Table E1 in the Online Repository at www.jacionline.org) for a specified food trigger plotted against the proportion of reported anaphylaxis reactions caused by that food (Fig 5). For Europe, crustaceans and cow’s milk appeared to cause a higher proportion of anaphylaxis in adults compared to peanut given the reported prevalence of allergy to those triggers. Hazelnut and some fruits caused a lower proportion of anaphylaxis for their reported prevalence compared to peanut; this could be due to their role as triggers for pollen–food allergy syndrome. Fish and crustaceans were common causes of anaphylaxis in adults in Asia, although this may be exaggerated by the relatively lower proportion of peanut anaphylaxis in this region.

## Discussion

As the food supply becomes increasingly globalized, there is a need to identify which foods should be singled out on food labels for disclosure in order to help protect food-allergic consumers. Epidemiologic data relating to prevalence and incidence of food allergy are limited by the impracticality of conducting food challenges in those with suspected allergy to distinguish between nonallergic adverse reactions to food, IgE sensitization without clinical reactivity, and true IgE-mediated food allergy with associated risk of anaphylaxis. For example, pollen–food allergy syndrome is thought to affect up to 35% of individuals in some regions,^75^ but such patients are considered to be at lower risk of anaphylaxis compared to those with primary food sensitization.^5^ In addition, the Codex requirements for allergen disclosure are for the scenario where the presence of the allergen may not be obvious (for example, in processed foods) rather than for fresh foods; because fruits and vegetables are generally visible and typically not consumed as highly processed foods, they do not currently feature as specified allergens in the Codex (although this may change in the future with the increased use of “vegetable protein concentrates”). To our knowledge, this analysis is the first in the literature to report a global assessment of the most common food triggers for anaphylaxis using a systematic approach. Rather than rely of reports of prevalence to specific food allergens that are limited by a lack of robust data,^4^ we instead used real-world data as to the most common causes of anaphylaxis presenting to medical facilities, as a surrogate measure to inform the choice of priority allergens for inclusion in legislation.

We found significant interregional and intraregional differences in the most common triggers for food anaphylaxis. Significant variations in the prevalence of allergy to different food triggers have been reported in Europe;^76^^,^^77^ it is therefore perhaps not surprising that similar differences were also evident for anaphylaxis, both within and between Codex regions. Peanut and tree nuts are a common cause of anaphylaxis in the European and NASWP regions, but less so in Asia. Wheat is generally less common as a cause of anaphylaxis, but it accounts for a disproportionate number of anaphylaxis presentations in China. These differences can potentially present a challenge for the regulation of food allergens within the supply chain, as food products produced and packaged in one country are often consumed in another; in addition, tourism can also significantly impact the specific food allergies that consumers might present with. In this respect, it is reassuring that in general, there was good agreement between local legislative requirements for allergen disclosure and the most common allergens causing anaphylaxis in that locality.

It was also revealing to compare the relative frequencies of food triggers causing anaphylaxis compared to their reported prevalence in causing food allergy. Data were available for this comparison for Europe, NASWP, and Asia. Using peanut as a reference allergen, our data indicate that crustaceans appear to cause a disproportionate number of anaphylaxis reactions in all 3 regions in adults. Interestingly, cow’s milk allergy also appears to cause a greater-than-expected proportion of anaphylaxis in children in Europe and Asia. Cow’s milk allergy may be considered to be a less serious food allergy, as it is commonly outgrown in early childhood. However, there are increasing data that in older children with persisting allergy to cow’s milk, it is a common cause of not just anaphylaxis but also near-fatal and fatal anaphylaxis.^11^^,^^12^^,^^16^ For example, in Greece, cow’s-milk allergy is relatively uncommon compared to the rest of Europe,^76^^,^^77^ yet it still accounts for around one quarter of anaphylaxis presentations.^18^ This may be due to a lower awareness of cow’s milk as a potential cause of severe reactions, as well as its ubiquitous use in Western-style diets, particularly in processed foods.

Conversely, at least in Europe, some fruits and tree nuts appeared to be less likely to cause anaphylaxis, presumably because these data do not distinguish between allergy due to primary food sensitization (with higher risk of anaphylaxis) and pollen–food allergy syndrome. Fruit as a food group was a common cause of anaphylaxis globally. However, the likely impact of differences in patterns of cross-sensitization and cross-reactivity are not obvious from these data. In Northern Europe, allergy to fruit is commonly associated with birch pollen sensitization; in Mediterranean regions, lipid transfer protein (LTP), particularly peach LTP, is also a common cause, which appears to be independent of pollen sensitization.^76^ However, in China, peach is also a relatively common cause of anaphylaxis, but this is usually associated with cross-reactivity to mugwort pollen; in contrast to European LTP allergy, LTP-related anaphylaxis in China is often due to primary sensitization to mugwort.^78^ More research is needed to better understand the clinical implications of geographic differences in sensitization patterns between different plant-derived allergens.

### Strengths and limitations of this study

The inclusion of global data sets identified through a systematic search of the literature is a key strength of this analysis. However, it is important to note the limitations of this analysis: different definitions were used to assign both anaphylaxis and the causative trigger, including International Classification of Disease (9th or 10th revision) codes, which are subject to miscoding.^79^ We believe that even with this limitation, the data would still represent the more severe end of the spectrum of allergic symptoms. The proportion of anaphylaxis due to any given specific food trigger is dependent on multiple factors, including underlying prevalence of allergy to that trigger within the population, consumption patterns, inherent ability of that allergen to cause more severe reactions, and host factors such as IgE sensitization. While these factors are all potential confounders, the use of real-world data provides an additional dimension to better understand which allergens are more likely to cause anaphylaxis than others. It is therefore not surprising that there is a clear correlation between prevalence of allergy to a specific food and the proportion of anaphylaxis cases it causes (Fig 3). This comparison was limited by the high uncertainty in data relating to food allergy prevalence and the very limited data from some regions. This is particularly a concern for North America, where challenge-based epidemiologic data are lacking; despite using systematic methodologies to estimate prevalence using household sampling approaches, allergy to cow’s milk in adults is apparently more common than peanut allergy (perhaps due to a lack of distinction between lactose intolerance and IgE-mediated allergy).^80^ The use of real-world anaphylaxis data may therefore provide less uncertainty as to the major causes of food anaphylaxis, compared to relying on estimates of food allergy prevalence alone.

### Conclusion

Using a systematic approach, we identified important and often region-specific differences in the most common food allergens causing anaphylaxis across the globe. However, legislative requirements for food allergen disclosure generally mirrored the local allergens most commonly responsible for food anaphylaxis events. Cow’s milk and shellfish/crustaceans are important causes of anaphylaxis globally, in addition to peanut and tree nuts. These data support the use of location-specific epidemiology to guide both public health policy and research with respect to food allergy.

**Clinical implications**

In addition to peanut and tree nuts, cow’s milk and shellfish/crustaceans are important causes of anaphylaxis globally.

## Figures and Tables

**Table E1:** Estimated prevalence of allergy to specific foods

Characteristic	Europe		North America/Southwest Pacific		Asia	
	Adults	Children	Adults	Children	Adults	Children
Reference	E3, E4	E3, E5	E6, E7	E7, E8, E9	E11, E12	E13, E14, E15, E16, E17
Peanut, % (95% CI)	0.35 (0.20-0.60)	0.42 (0.25-0.70)	1.8 (0.6-1.9)	2.60 (2.17-3.11)	0.46 (0.36-0.58)	0.21 (0.17-0.27)
Tree nuts (combined), % (95% CI)			1.2 (1.1-1.3)	1.77 (1.26-2.47)		0.12 (0-6.63)
Cashew, % (95% CI)		0.11 (0-0.62)	0.5 (0.5-0.6)	1.04 (0.59-1.84)		
Hazelnut, % (95% CI)	0.86 (0.39-1.90)	0.28 (0.10-0.77)	0.6 (0.5-0.7)	0.61 (0.54-0.69)		
Walnut, % (95% CI)	0.30 (0.14-0.66)	0.12 (0.04-0.41)	0.6 (0.6-0.7)	0.61 (0.54-0.69)		
Sesame, % (95% CI)	0.01 (0-1.35)	0.07 (0.01-0.88)	0.2 (0.2-0.3)	0.21 (0.17-0.25)		0.07 (0.05-0.10)
Mustard, % (95% CI)	0.00 (0-2.29)					
Wheat, % (95% CI)	0.09 (0.03-0.29)	0.16 (0.09-0.29)	0.8 (0.7-0.9)	0.23 (0.10-0.53)	0.14 (0.04-0.53)	0.06 (0.02-0.23)
Buckwheat, % (95% CI)		0.03 (0-0.37)				0.16 (0.13-0.21)
Hen’s egg, % (95% CI)	0.08 (0.03-0.25)	0.32 (0.17-0.60)	0.8 (0.7-0.9)	0.94 (0.53-1.67)	0.30 (0.22-0.40)	0.18 (0.04-0.80)
Cow’s milk, % (95% CI)	0.16 (0.07-0.35)	0.35 (0.20-0.63)	1.1 (0.9-2.1)∗	0.79 (0.26-2.33)	0.48 (0.38-0.61)	0.07 (0.01-0.82)
Celery, % (95% CI)	0.14 (0.04-0.45)	0.04 (0-1.50)				
Shrimp/crustaceans, % (95% CI)	0.41 (0.21-0.80)	0.11 (0.04-0.29)	1.9 (1.8-2.1)	0.72 (0.36-1.45)	0.27 (0-7.5)	0.55 (0.36-0.84)
Fish, % (95% CI)	0.14 (0.06-0.33)	0.04 (0.01-0.18)	0.9 (0.8-1.0)	0.52 (0.24-1.12)	0.22 (0.01-1.4)	0.29 (0.26-0.32)
Soybean, % (95% CI)	0.04 (0.01-0.19)	0.19 (0.07-0.57)	0.6 (0.5-0.7)	0.20 (0.06-0.66)		0.07 (0.06-0.09)
Lentil, % (95% CI)	0.04 (0.01-0.35)	0.10 (0.01-0.79)				
Peach, % (95% CI)	0.64 (0.24-1.67)	0.40 (0.20-0.80)				
Kiwi, % (95% CI)	0.61 (0.32-1.17)	0.49 (0.28-0.83)				0.02 (0-0.70)
Banana, % (95% CI)	0.12 (0.02-0.71)	0.33 (0.13-0.85)				
Apple, % (95% CI)	0.67 (0.30-1.51)	0.65 (0.31-1.36)				
Carrot, % (95% CI)	0.49 (0.27-0.91)	0.24 (0.11-0.51)				

For further details, see this article’s Supplementary Methods.

∗ Prevalence based on Canadian data.

## Methods

### Estimated prevalence of allergy to specific foods

Prevalence rates for individual allergens were pooled across included studies using a generalized linear mixed model in R 4.0.3 software (R Foundation for Statistical Computing, Vienna, Austria; https://www.r-project.org/) (metaprop function, metafor package, logit transformation with a random intercept logistic regression model for the summary estimate, with a continuity correction of 0.5). This approach avoids many of the issues surrounding the use of transformations when undertaking meta-analyses of proportions.E1, E2 We conducted meta-analysis even if significant heterogeneity was seen between study estimates, as is the norm when conducting meta-analysis of proportions. Normal approximation was used for calculating confidence intervals.

For Europe, prevalence was estimated on the basis of reported rates of challenge-positive food allergy reported in a systematic review^E3^ and also generated by the EuroPrevall studies (on the basis of study-defined probable food allergy).E4, E5

For the North America/Southwest Pacific (NASWP) region, there are no prevalence data for adults that are based on food challenges in unselected populations. Instead, adult prevalence data were extracted from Gupta et al^E6^ and compared to equivalent data for Canada.^E7^ As outlined in the Discussion, as a result of concerns that the reported prevalence of cow’s-milk allergy in adults by Gupta et al is likely to be an overestimate, the equivalent figure for Canada was used instead. For prevalence of food allergy in children in the NASWP region, rates were pooled from studies reporting prevalence for the United States,^E8^ Canada,^E7^ and Australia^E9^ (only the latter incorporated food challenges to assess prevalence).

Limited data exist for the prevalence of food allergy in the Asia region.^E10^ For adults, data were pooled from studies conducted in Taiwan^E11^ and India.^E12^ For children, data for China and India were extracted from the EuroPrevall-INCO Surveys^E13^ and 2 studies from ThailandE14, E15 (all of which included food challenges to confirm food allergy), as well as published data for Japan^E16^ and Korea^E17^ that did not rely on challenge-positive outcomes.

The pooled estimates for reported prevalence to specific food allergens are shown in Table E1.
